# Effect of Timing of Mechanical Chest Compression Device Applied on Adrenaline Time: A Single-Center Prospective Cohort Study

**DOI:** 10.7759/cureus.91022

**Published:** 2025-08-26

**Authors:** Shingo Yamazaki, Koshi Nakagawa, Hiroki Ueta, Hideharu Tanaka

**Affiliations:** 1 Emergency Medical Services Division, Yokosuka City Fire Bureau, Yokosuka, JPN; 2 Graduate School of Emergency Medical System, Kokushikan University, Tokyo, JPN; 3 Department of Integrated Science and Engineering for Sustainable Societies, Faculty of Science and Engineering, Chuo University, Tokyo, JPN; 4 Research Institute of Disaster Management and Emergency Medical System, Kokushikan University, Tokyo, JPN

**Keywords:** adrenaline, advanced life support, cardiopulmonary resuscitation, emergency medical services, mechanical chest compression device, out-of-hospital cardiac arrest

## Abstract

Objectives:* *The relationship between the timing of mechanical chest compression device (MCD) application and adrenaline administration in out-of-hospital cardiac arrest (OHCA) remains unclear. This study aimed to examine the association between the time from patient contact to MCD chest compression initiation (MCD time) and that from patient contact to adrenaline administration (adrenaline time) in OHCA, in order to assess whether early MCD application facilitates early adrenaline administration.

Methods:* *This single-center prospective cohort study used emergency transport data from the Yokosuka City Fire Bureau. Cases where adrenaline was administered by emergency life-saving technicians after MCD application in prehospital settings were included. Patients were categorized into the early (1-5 min) and late (≥ 6 min) MCD groups based on the median MCD time. The primary outcome was adrenaline time, and the secondary outcome was scene time interval (STI), defined as the time from patient contact to departure from the scene. Based on a median STI of 16 min, cases were categorized into short (1-16 min) and long (≥ 17 min) groups. Multivariable logistic regression analysis was conducted using propensity scores to adjust for confounders.

Results: Overall, 174 patients were analyzed, with 92 (52.9%) and 82 (47.1%) in the early and late MCD groups, respectively. The early MCD group had a significantly shorter median adrenaline time than the late MCD group (14.0 vs. 18.0 min). Early MCD application was significantly associated with earlier adrenaline administration (adjusted odds ratio (AOR): 3.47; 95% confidence interval (CI): 1.61-7.49) but not with reduced STI (AOR: 1.71; 95% CI: 0.80-3.68).

Conclusions: Early MCD application in OHCA was associated with earlier adrenaline administration without extending STI. Regional Medical Control Councils should establish protocols and conduct simulation training to optimize early MCD application and early adrenaline administration.

## Introduction

Approximately 130,000 out-of-hospital cardiac arrest (OHCA) cases occur annually in Japan. Despite an increasing trend in the survival proportion of OHCA patients over time, overall survival remains low [1]. Early intervention by emergency medical service (EMS) personnel can significantly improve outcomes for OHCA patients [2,3]. Early adrenaline administration is significantly associated with both survival and favorable neurological outcomes after OHCA [4-7].

The Japan Resuscitation Council Guideline 2020 strongly recommends administering adrenaline as soon as feasible for a non-shockable cardiac rhythm. For patients with a shockable rhythm, the guideline suggests adrenaline administration after unsuccessful initial defibrillation attempts [8]. These recommendations underscore the crucial role of adrenaline in OHCA management.

Resuscitation strategies for OHCA differ between Japan and Western countries. Western countries prioritize achieving the return of spontaneous circulation (ROSC) at the scene [9]; however, in Japan, the focus is on performing cardiopulmonary resuscitation (CPR) while rapidly transporting patients to the nearest emergency department [10]. In Japan, two EMS personnel must perform continuous CPR in OHCA cases, while another administers advanced life support (ALS), including adrenaline administration, and prepares for transport. Pumper-ambulance collaboration is typically used to secure additional personnel. However, in confined spaces, such as narrow scenes or inside an ambulance, this collaboration may hinder emergency operations. Consequently, critical interventions, such as early adrenaline administration, may be delayed.

Mechanical chest compression devices (MCDs) exhibit no significant difference in survival proportion compared to manual CPR in OHCA [11-13]. However, in Japanese prehospital settings, where limited EMS personnel must simultaneously perform rapid transport and ALS, MCDs are crucial for addressing personnel shortages and complex transportation [14]. Their application frees up one EMS personnel from performing manual CPR, enabling the other two EMS personnel to efficiently prepare for ALS, which increases the likelihood of early adrenaline administration [14-17].

Currently, few protocols in Japan dictate the application of MCDs, making it a subjective decision for emergency life-saving technicians (ELSTs). Therefore, analyzing the optimal timing for MCD application to facilitate early adrenaline administration can aid in developing standardized protocols.

This study aimed to clarify the relationship between the time from patient contact to the initiation of MCD chest compressions and the time from patient contact to adrenaline administration in OHCA cases where adrenaline was administered after MCD application.

## Materials and methods

Study design and ethical considerations

This was a single-center prospective cohort study of OHCA cases registered in the emergency transport data of the Yokosuka City Fire Bureau from May 1, 2022, to February 29, 2024, and approved by the Ethics Committee at Kokushikan University (approval number: 23034). The requirement for written informed consent was waived owing to the use of anonymized data. To minimize bias from the Hawthorne effect, EMS personnel were not informed of the study [18].

We defined the MCD time as the time from patient contact to the initiation of mechanical chest compressions and the adrenaline time as the time from patient contact to adrenaline administration.

Emergency medical services systems in Yokosuka City Fire Bureau

In April 2017, the Yokosuka City Fire Bureau began operating as a wide-area fire department covering the cities of Yokosuka and Miura in Kanagawa Prefecture, Japan. As of February 2024, the jurisdiction covered an area of 132.86 km2 and had a population of 413,835. The Bureau operates 14 ambulances 24 h a day, 7 d a week, with an additional daytime ambulance available from 8:30 am to 5:15 pm to meet daytime emergency demands, and has one dispatch center. The number of patients transported by EMS in 2023 was 30,087. Upon receiving a 119-emergency call (Japan's universal emergency number for fire and ambulance services), the dispatch center deploys the nearest ambulance to the scene for hospital transport. A pumper-ambulance collaboration is implemented in all cases of suspected OHCA based on the emergency call. In this system, four non-ELST-certified firefighters are dispatched in a pumper vehicle alongside EMS personnel to support emergency operations. This collaboration occurs when the ambulance response time is expected to be long or when additional support is requested on the scene. For example, when OHCA is confirmed upon EMS arrival or when the patient is in a challenging location, including upper floors, underground levels, or confined spaces [19]. If an OHCA is suspected based on an emergency call, telecommunicator assistance is provided to enable the caller to perform CPR [19].

Each ambulance has three EMS personnel, including at least one ELST qualified to provide ALS, including intravenous access, adrenaline administration, and advanced airway management such as endotracheal intubation and supraglottic airway devices, for OHCA patients.

Since April 2006, specially trained ELSTs have been authorized to administer adrenaline under protocols established by the Miura Peninsula Regional Medical Control Council and online medical direction by physicians. The protocol allows adrenaline administration for OHCA patients aged ≥ 8 years, with no limit on the number of doses. If ventilation via a bag-valve mask is sufficient, priority is given to intravenous access and adrenaline over advanced airway management. Adrenaline administration must not prolong the scene time interval (STI), defined as the time from patient contact to departure from the scene. In Japan, EMS personnel are not authorized to terminate resuscitation in the field, and all patients in whom resuscitation is attempted are transported to the hospital. However, exceptions include OHCA patients showing obvious signs of death, such as decapitation, severe traumatic brain injury with cerebral prolapse, skeletonization, or early postmortem changes, who are not transported to the hospital.

The challenging terrain of the Bureau jurisdiction, with its many valleys and hills, complicates the delivery of high-quality CPR. Therefore, all ambulances are equipped with the Lund University Cardiopulmonary Assist System 3 (LUCAS3) chest compression device (Stryker Corporation, Kalamazoo, MI, USA). When OHCA is suspected, EMS personnel bring the LUCAS3 to the scene. However, its use is not governed by a specific protocol and is left to the discretion of the ELSTs. Typically, EMS personnel apply the LUCAS3, and patient transport is facilitated using a Ferno Scoop EXL Stretcher (Ferno-Washington, Inc., Wilmington, OH, USA).

Inclusion and exclusion criteria

The detailed inclusion and exclusion criteria are presented in Table 1. This study included OHCA cases that occurred within the jurisdiction of the Yokosuka City Fire Bureau from May 1, 2022, to February 29, 2024, in which adrenaline administration was performed by an ELST after MCD application in prehospital settings. Exclusion criteria were as follows: (i) patients aged < 8 years in whom adrenaline administration was not indicated; (ii) achievement of ROSC before EMS personnel contact; (iii) ALS performed by physicians; (iv) cardiac arrest cases witnessed by physicians, EMS personnel, or firefighters; and (v) discontinued MCD application or a switch to manual chest compressions.

**Table 1:** Inclusion and exclusion criteria ALS: advanced life support; ELST: emergency life-saving technicians; EMS: emergency medical service; MCD: mechanical chest compression device; OHCA: out-of-hospital cardiac arrest; ROSC: return of spontaneous circulation

Category	Criteria
Inclusion criteria	OHCA cases occurring in the jurisdiction of the Yokosuka City Fire Bureau between May 1, 2022, and February 29, 2024, in which adrenaline was administered by an ELST following MCD application in the prehospital setting.
Exclusion criteria	(i) Patients aged < 8 years in whom adrenaline administration was not indicated.
	(ii) Achievement of ROSC before EMS personnel contact.
	(iii) ALS performed by physicians.
	(iv) Cardiac arrest witnessed by physicians, EMS personnel, or firefighters.
	(v) discontinued MCD application or a switch to manual chest compressions.

Participants were categorized into the following two groups based on the median MCD time of 5 min: early (1-5 min) and late MCD (≥ 6 min) groups.

Sample size and study duration

This study was not based on calculated sample sizes. Instead, it was conducted over 22 months, from May 1, 2022, to February 29, 2024, owing to practical time constraints on the feasible research duration.

Outcomes

The primary and secondary outcomes were the adrenaline time (min) and STI (min), respectively. Adrenaline time was categorized into early (1-17 min) and late (≥ 18 min) administration groups based on a median adrenaline time of 17 min. Converting continuous variables into categorical variables may lead to information loss and reduced model accuracy [20]. However, considering the difficulties in time management during emergency operations, setting the adrenaline time at or below the median provides a tangible target. Multiple regression analysis can be used to evaluate the temporal relationship between the MCD and adrenaline time. However, determining a clear target time for MCD application in actual emergency operations is difficult. Therefore, we aimed to determine the specific timing for the application of MCD suitable for early adrenaline administration by categorizing the adrenaline time. Based on a median STI of 16 min, the STI was categorized into short (1-16 min) and long (≥ 17 min) groups. The Miura Peninsula Regional Medical Control protocol requires early adrenaline administration while minimizing STI. Therefore, our study evaluated this aspect as a secondary outcome.

Data variables

Patient-level variables used in this study were as follows: (i) patient demographics: age (< 65 or ≥ 65 years) and sex (male or female); (ii) cardiac arrest event characteristics: location (private residence or public location), floor level (ground level/higher floors), season (spring (March to May), summer (June to August), autumn (September to November), or winter (December to February)), etiology (cardiogenic or non-cardiogenic), and witness status (witnessed or unwitnessed); (iii) bystander interventions: bystander CPR (yes or no) and public access defibrillation (yes or no); (iv) EMS factors: number of ELSTs in the ambulance (one, two or three), initial rhythm (ventricular fibrillation or pulseless ventricular tachycardia (shockable rhythm), pulseless electrical activity or asystole (non-shockable rhythm)), defibrillation (yes or no), advanced airway management (yes or no), number of intravenous access (single or two), location of MCD application (on-scene or into the ambulance), and pumper-ambulance collaboration (yes or no); and (v) interval: emergency call-to-patient contact interval (response time interval; < 11 min or ≥ 11 min), STI (< 17 min or ≥ 17 min), departure from the scene-to-arrival at the emergency department interval (transport time interval; < 10 min or ≥ 10 min), adrenaline time (< 18 min or ≥ 18 min), MCD time (< 6 min or ≥ 6 min), and MCD back plate insertion-to-MCD chest compression initiation interval (MCD setup interval; < 30 s or ≥ 30 s). The MCD preparation interval was measured using wristwatches or similar devices by EMS personnel. Age, response time interval, STI, transport time interval, and MCD setup interval were continuous variables that did not satisfy the assumption of linearity. Therefore, they were categorized before being input into the analysis model. The analysis excluded cases of public access defibrillation owing to their small number to prevent unstable outcomes.

Statistical analysis

For patient characteristics, continuous variables are shown as medians (interquartile ranges (IQR)), and categorical variables are presented as numbers (%).

First, we created a scatter plot of the MCD and adrenaline time, and then calculated Pearson’s correlation coefficient. Second, to analyze the association between the MCD time and the adrenaline time, we conducted a multivariable logistic regression analysis using the propensity score as a covariate. To calculate the propensity score, we included the following variables that could influence the early application of MCD: age, sex, location, floor level, season, etiology, witness status, bystander interventions, EMS factors (number of ELSTs in the ambulance, initial rhythm, defibrillation, advanced airway management, location of the MCD application, and pumper-ambulance collaboration), and time data (response time interval, STI, and transport time interval). As the propensity score did not meet the linearity assumption, it was categorized into five groups with 0.2 increments [21]. We used multivariable logistic regression analysis, with the propensity score and number of intravenous access as covariates, to estimate the adjusted odds ratio (AOR) and 95% confidence interval (CI) for the association between the MCD and adrenaline time. In multivariable logistic regression, including more than one-tenth of the number of events in the least frequent outcome category can lead to overfitting, resulting in reduced analysis accuracy [22]. Therefore, we used the propensity score as a covariate in the analysis. In this study, 18 variables were selected as covariates. However, owing to the low event occurrence rate (82 events) in the late administration group, including all variables was deemed impractical [23]. Therefore, propensity scores were used as covariates to maintain analytical accuracy and prevent overfitting. Secondary outcomes were analyzed using the same method as that for the primary outcome. A two-sided significance level of 0.05 (two-sided) was used for all analyses. Statistical analyses were performed using JMP Pro version 15 (SAS Institute Inc., Cary, NC, USA).

## Results

Flowchart of out-of-hospital cardiac arrest (OHCA) patients

**Figure 1:**
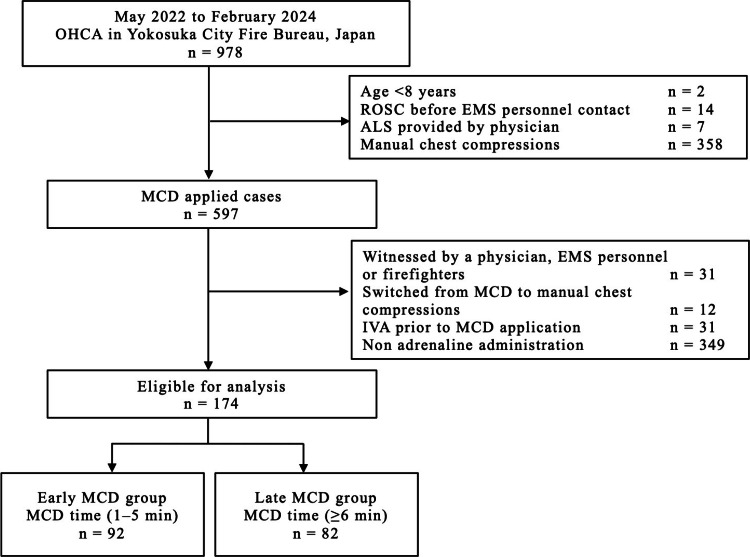
Inclusion and exclusion criteria for participant selection and enrollment in the study. ALS: advanced life support; CPR: cardiopulmonary resuscitation; EMS: emergency medical service; IVA: intravenous access; MCD: mechanical chest compression device; OHCA: out-of-hospital cardiac arrest; ROSC: return of spontaneous circulation; MCD time, the time from patient contact to the initiation of MCD chest compressions

The study flowchart, showing the included and excluded OHCA cases, is presented in Figure 1. During the study period, 978 OHCA cases were registered, of which 597 (61.0%) were treated with MCD. This study included 174 patients who were administered adrenaline after MCD application. Of these, 92 (52.9%) and 82 (47.1%) patients were in the early and late MCD groups, respectively.

Patient characteristics

Patient characteristics are shown in Table 2. The late MCD group had an increased proportion of OHCA occurrences in private residences (early vs. late: 66 (71.7%) vs. 67 (81.7%)). The early MCD group had an increased proportion of OHCA occurrences at ground level (early vs. late; 71 (77.2%) vs. 46 (56.1%)). The early MCD group had an elevated proportion of MCD applied on-scene (early vs. late: 88 (95.7%) vs. 48 (58.5%)). The median (IQR) of the STI was 15.5 (11.2-19.0) and 17.0 (13.0-20.0) min in the early and late MCD groups, respectively. The median (IQR) of the adrenaline time was 14.0 (12.0-19.0) and 18.0 (15.7-22.2) min in the early and late MCD groups, respectively. The median (IQR) of the MCD time was 3.0 (2.0-4.0) and 8.0 (7.0-10.0) min in the early and late MCD groups, respectively.

**Table 2:** Characteristics of out-of-hospital cardiac arrest patients who received treatment with a mechanical chest compression device AAM: advanced airway management; CPR: cardiopulmonary resuscitation; EMS: emergency medical service; ELST: emergency life-saving technician; IVA: intravenous access; IQR: interquartile range; MCD: mechanical chest compression device; PAD: public access defibrillation Response time interval, emergency call-to-patient contact interval; scene time interval, patient contact-to-departure from the scene interval; transport time interval, departure from the scene-to-arrival at emergency department interval.

Characteristics	All Cases	Early MCD Group	Late MCD Group
n(%)	174 (100.0)	92 (52.9)	82 (47.1)
Age, years; median (IQR)	80.0 (72.0-86.0)	82.0 (74.2-87.7)	79.0 (70.0-84.0)
≥65	149 (85.6)	81 (88.0)	68 (82.9)
Sex, male	122 (70.1)	64 (69.6)	58 (70.7)
Location			
Private residence	133 (76.4)	66 (71.7)	67 (81.7)
Public location	41 (23.6)	26 (28.3)	15 (18.3)
Floor level			
Ground level	117 (67.2)	71 (77.2)	46 (56.1)
Higher floors	57 (32.8)	21 (22.8)	36 (43.9)
Season			
Spring (March to May)	30 (17.2)	15 (16.3)	15 (18.3)
Summer (June to August)	33 (19.0)	15 (16.3)	18 (22.0)
Autumn (September to November)	41 (23.6)	29 (31.5)	12 (14.6)
Winter (December to February)	70 (40.2)	33 (35.9)	37 (45.1)
Etiology			
Cardiogenic	128 (73.6)	67 (72.8)	61 (74.4)
Non-cardiogenic	46 (26.4)	25 (27.2)	21 (25.6)
Witnessed	49 (28.2)	26 (28.3)	23 (28.0)
Bystander CPR	78 (44.8)	42 (45.7)	36 (43.9)
PAD	1 (0.6)	1 (1.1)	0 (0.0)
Number of ELSTs in the ambulance			
One	62 (35.6)	36 (39.1)	26 (31.7)
Two or three	112 (64.4)	56 (60.9)	56 (68.3)
Initial rhythm			
Shockable	13 (7.5)	8 (8.7)	5 (6.1)
Non-shockable	161 (92.5)	84 (91.3)	77 (93.9)
Defibrillation by EMS	23 (13.2)	13 (14.1)	10 (12.2)
AAM	141 (81.0)	78 (84.8)	63 (76.8)
Number of IVA			
Single	159 (91.4)	85 (92.4)	74 (90.2)
Two	15 (8.6)	7 (7.6)	8 (9.8)
Location of the MCD application			
On-scene	136 (78.2)	88 (95.7)	48 (58.5)
In the ambulance	38 (21.8)	4 (4.3)	34 (41.5)
Pumper-ambulance collaboration	165 (94.8)	88 (95.7)	77 (93.9)
Time data			
Response time interval, median (IQR), min	10.0 (8.0,12.0)	10.0 (8.25,12.0)	10.0 (8.0,12.0)
Scene time interval, median (IQR), min	16.0 (13.0,20.0)	15.5 (11.2,19.0)	17.0 (13.0,20.0)
Transport time interval, median (IQR), min	9.0 (7.0,12.0)	9.0 (7.0,12.0)	10.0 (6.0,13.0)
Contact to adrenaline administration, median (IQR), min	17.0 (13.0,20.2)	14.0 (12.0,19.0)	18.0 (15.7,22.2)
Contact-to-MCD chest compression initiation, median (IQR), min	5.0 (3.0,8.0)	3.0 (2.0,4.0)	8.0 (7.0,10.0)
MCD back plate insertion-to-MCD chest compression initiation, median (IQR), s	30.0 (20.0,52.5)	30.0 (20.0,60.0)	30.0 (20.0,46.2)

Correlation between mechanical chest compression device (MCD) time and adrenaline time

**Figure 2:**
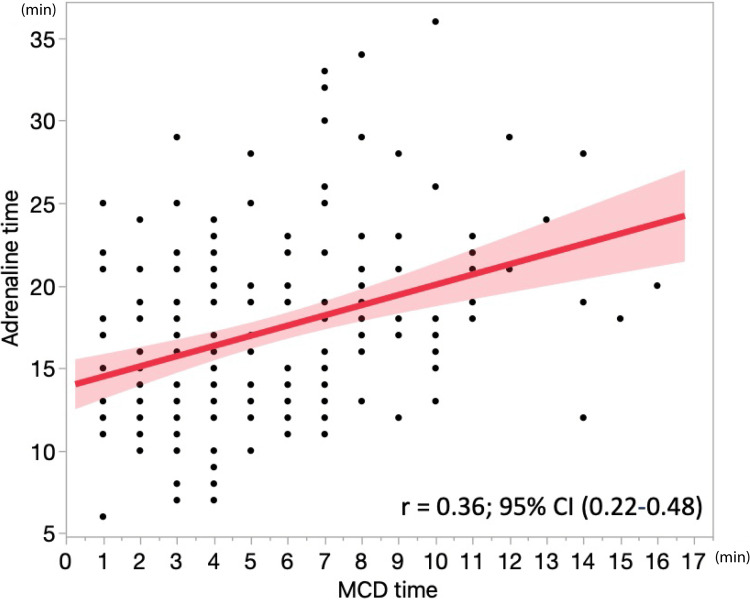
Correlation between MCD time and adrenaline time. CI: confidence interval; MCD: mechanical chest compression device; r: correlation coefficient Adrenaline time, the time from patient contact to adrenaline administration; MCD time, the time from patient contact to the initiation of MCD chest compressions.

A scatter plot illustrating the correlation between the MCD time and the adrenaline time is shown in Figure 2. A significant weak positive correlation was found between the MCD time and the adrenaline time (r = 0.36, p = 0.001).

Association between the early MCD group and adrenaline time

The results of multivariable logistic regression analysis evaluating the association between the early MCD group and the adrenaline time are presented in Table 3. Compared to the late MCD group, the early MCD group was significantly associated with the early administration group (AOR: 3.47; 95% CI: 1.61-7.49).

**Table 3:** Results of multivariable logistic analysis of MCD time and adrenaline time with propensity score as a covariate a) Adjusted variables: propensity score (stratified into five groups in 0.2 increments), number of intravenous accesses, b) Propensity scores were calculated using the following variables (age, sex, location, floor level, season, etiology, witness status, bystander interventions, number of emergency life-saving technicians in the ambulance, initial rhythm, emergency medical service treatment (defibrillation, advanced airway management), location of the MCD application, pumper-ambulance collaboration, and interval data (emergency call-to-patient contact interval, patient contact-to-departure from the scene interval, and departure from the scene-to-arrival at emergency department interval), c) area under the receiver operating characteristic curve =0.72 AOR: adjusted odds ratio; CI: confidence interval; COR: crude odds ratio; MCD: mechanical chest compression device. MCD time, the time from patient contact to initiation of mechanical chest compression; adrenaline time, the time from patient contact to adrenaline administration.

MCD Time	Adrenaline Time (≤ 17 min)	
	COR: (95% CI)	AOR ^a)–c)^: (95% CI)
Early MCD Group	3.79 (2.02–7.11)	3.47 (1.61–7.49)
Late MCD Group	Reference	Reference

Association between the early MCD group and STI

The results of the multivariable logistic regression analysis evaluating the association between the early MCD group and the STI are shown in Table 4. Compared to the late MCD group, the early MCD group was not significantly associated with a reduction in the STI (AOR: 1.71; 95% CI: 0.80-3.68).

**Table 4:** Results of multivariable logistic analysis of MCD time and STI with propensity score as a covariate a) Adjusted variables: propensity score (stratified into five groups in 0.2 increments), b) Propensity scores were calculated using the following variables (age, sex, location, floor level, season, etiology, witness status, bystander interventions, number of emergency life-saving technicians in the ambulance, initial rhythm, emergency medical service treatment (defibrillation, advanced airway management, number of intravenous access), location of the MCD application, pumper-ambulance collaboration, interval data (patient contact to adrenaline administration, MCD back plate insertion-to-MCD chest compression initiation, emergency call-to-patient contact, and departure from the scene-to-arrival at emergency department intervals), c) area under the receiver operating characteristic curve=0.64 AOR: adjusted odds ratio; CI: confidence interval; COR: crude odds ratio; MCD: mechanical chest compression device. MCD time, the time from patient contact to initiation of mechanical chest compression; STI, the time from patient contact to departure from the scene.

MCD Time	Scene Time Interval (≤ 16 min)	
	COR: (95% CI)	AOR ^a)–c)^: (95% CI)
Early MCD group	1.49 (0.81–2.71)	1.71 (0.80–3.68)
Late MCD group	reference	Reference

## Discussion

This study examined the association among the MCD time, adrenaline time, and STI in patients who were administered adrenaline after MCD application in the Yokosuka City Fire Bureau, Japan. We found a significant association between early MCD application and early adrenaline administration, but not between early MCD application and STI reduction. A comparison between the early and late MCD groups revealed that the median MCD time was 5 min shorter in the early group than in the late group. Furthermore, the median adrenaline time was 4 min shorter in the early group than in the late group, likely due to the early switch from manual chest compressions to MCD, which eliminated the need for manual chest compressions by EMS personnel. This effectively had the same impact as increasing the number of EMS personnel by one, enabling early adrenaline administration. This result supports a previous finding [14], suggesting that MCD improves the quality of CPR and increases the efficiency of emergency operations by compensating for personnel shortages. This study provides an effective solution to space constraints and personnel shortages in the Japanese EMS. Early MCD application compensates for personnel limitations and facilitates early adrenaline administration in confined spaces, improving the quality of emergency operations for OHCA patients, regardless of the operational environment.

The median setup time for MCD was 30 s. Although continuous chest compressions are crucial in the early stages of cardiac arrest [24], early adrenaline administration is important to improve resuscitation outcomes in OHCA patients. Therefore, it is important to reduce the MCD setup time through team training [25,26] and ensure early adrenaline administration. Future studies should include comprehensive regional data analysis to enable comparative studies with other facilities and the development of standardized protocols for resuscitation strategies using MCD.

In evaluating STI, the early MCD group showed a non-significant 1.5-min reduction trend. This suggests that early MCD application may facilitate early adrenaline administration without significantly prolonging the STI. To achieve both early MCD application and shortening of the STI, it is crucial to establish effective transport methods for confined spaces where the use of scoop stretchers is limited. This requires selecting appropriate transportation equipment, including a combination of an MCD and a carry sheet [27], and considering their proper usage. However, further evaluation is necessary because of the risk of chest compression position shifting [28] when using equipment that does not stabilize the patient's back. In cases where early MCD application is difficult in emergency scenes, persisting with its use may unnecessarily prolong the STI. Therefore, simulation training that considers the conditions of emergency scenes is essential for effective MCD application.

This study has some limitations. First, the analysis was limited to data from the Yokosuka City Fire Bureau, restricting the generalizability of the results. Furthermore, owing to differences in EMS systems and resuscitation strategies for OHCA across countries, careful consideration is needed when applying these findings to different prehospital settings. Second, the absence of an established protocol for MCD use in Yokosuka City Fire Bureau prevents the identification of factors that determine the timing of MCD application by ELSTs. Third, although MCD application is primarily performed by EMS personnel, the involvement of firefighters in the MCD application has not been evaluated when a pumper-ambulance collaboration is in place, which could potentially affect the MCD time. Fourth, this study focused on the adrenaline time and could not address the relationship with patient outcomes. Fifth, the small sample size of this study limited the precision of the analysis. Therefore, future research with larger sample sizes should be conducted to analyze the impact on patient outcomes.

## Conclusions

Early MCD application in OHCA cases was significantly associated with early adrenaline administration without extending the STI. This finding supports the strategic use of MCDs to facilitate prompt drug administration with limited EMS personnel, while maintaining efficient emergency operations.

In the future, Regional Medical Control Councils should enhance prehospital care quality by establishing standardized protocols that integrate early MCD application with early adrenaline administration and implementing practical simulation-based training programs.
